# Association between controlling nutritional status score and the prognosis of patients with heart failure: a systematic review and meta-analysis

**DOI:** 10.3389/fcvm.2025.1665713

**Published:** 2025-10-20

**Authors:** Yuan Yuan, Su-Ping Wang, Yi Guan, Qing-Yi Yang, Peng-Yu Zhong, Hao-Yu Wang

**Affiliations:** Department of Cardiology, The Second Clinical Medical College of North Sichuan Medical College, Nanchong, China

**Keywords:** CONUT, heart failure, prognosis, malnutrition, all-cause mortality

## Abstract

**Background:**

Malnutrition frequently complicates heart failure (HF), interacting with systemic inflammation, metabolic dysregulation, and immune dysfunction to accelerate disease progression. The Controlling Nutritional Status (CONUT) score, derived from objective laboratory parameters (serum albumin, total cholesterol, lymphocyte count), quantifies nutritional derangements and has emerged as a promising tool for HF risk stratification and prognostic prediction. However, accumulating evidence requires systematic synthesis to establish its clinical validity.

**Methods:**

A comprehensive literature search was conducted in databases including PubMed, Embase, the Cochrane Library and Web of Science, covering all available records up to January 27, 2025, to identify research examining the association between the CONUT score and HF outcomes.

**Results:**

The analysis included 28 cohort studies. Pooled data demonstrated a significant correlation between elevated CONUT scores and higher rates of all-cause mortality (HR = 1.57, 95% CI 1.35–1.83; *P* < 0.00001). Despite substantial heterogeneity, sequential exclusion sensitivity analyses confirmed the robustness of this association, with recalculated estimates consistently showing overlapping confidence intervals across all analytical scenarios.

**Conclusion:**

Based on the definition of the CONUT score, malnutrition remains a significant factor associated with overall mortality risk in individuals diagnosed with heart failure, even after controlling for potential confounders. Utilizing the CONUT score for nutritional assessment enables clinicians to detect patients who are more likely to experience unfavorable clinical outcomes.

**Systematic Review Registration:**

https://www.crd.york.ac.uk/PROSPERO/view/CRD420251023217, PROSPERO CRD420251023217.

## 1 Introduction

Heart failure constitutes the final phase in the progression of various cardiovascular diseases. Despite significant advances in heart failure management, its incidence continues to rise (1). Epidemiological studies indicate that heart failure affects an estimated 64.3 million individuals globally, with a prevalence of 4%–11% among individuals aged ≥65 (2). As reported by the Global Burden of Disease Study, the age-standardized mortality rate attributed to heart failure increased by 38.3% between 1990 and 2017, and the condition accounted for approximately 3 million deaths globally in 2019 (3). This growing disease burden is closely linked to population aging, improved survival rates of coronary heart disease, and the growing prevalence of metabolic disorders. Nutritional deficiencies are widespread in heart failure patients: 24% of chronic heart failure (CHF) patients exhibit hypoalbuminemia (<3.5 mg/dl), and 68% experience muscle wasting (4). However, current heart failure guidelines offer limited guidance on managing nutritional disorders (5). Research suggests that the pathogenesis of heart failure is associated with novel inflammation-related circulating biomarkers (6). Malnutrition accelerates disease progression by promoting muscle catabolism, immune dysfunction, and exacerbated inflammatory responses (5).

The CONUT Score integrates three objective biochemical parameters-serum albumin, total cholesterol, and peripheral blood lymphocyte count (Table 1), to provide a multidimensional quantitative assessment system for nutritional status. Scores on the CONUT scale span from 0–12, with elevated values signifying poorer nutritional status. Recent clinical research has shown a significant link between elevated CONUT scores and adverse clinical outcomes among individuals with heart failure (HF). For example, Kato et al., in a multicenter cohort analysis (7), found that individuals with a CONUT score ≥5 had a 2.80-fold increased risk of all-cause mortality compared to controls (HR = 2.80, 95% CI 1.92–4.08). Similarly, Zhao et al. found (8) that a higher CONUT score at admission independently predicted poor prognosis in individuals with systolic heart failure (HR = 1.79, 95% CI 1.37–2.32). The underlying mechanisms may involve malnutrition-induced multifaceted pathophysiological alterations. However, systematic evidence supporting the prognostic value of the CONUT score in HF remains limited and warrants further synthesis.

**Table 1:** CONUT score calculation criteria.

Parameter	Score			
Serum Albumin (g/L)	≥35	30–34.9	25–29.9	<25
Albumin Score	0	2	4	6
Total Cholesterol (mg/dl)	≥180	140–179	100–139	<100
Cholesterol Score	0	1	2	3
Lymphocyte(10^9^/L)	≥1.60	1.20–1.59	0.80–1.19	<0.80
Lymphocyte Score	0	1	2	3

Although numerous observational studies indicate a potential link between the CONUT score and adverse prognosis in heart failure patients, its effectiveness as a predictive tool still lacks robust evidence-based support. This meta-analysis therefore aims to investigate the relationship between the CONUT score and clinical outcomes in heart failure patients, thereby providing a foundation for improved clinical management and risk stratification.

## 2 Methods

This meta-analysis was performed following the guidelines of the PRISMA statement and was prospectively registered in the PROSPERO database (CRD420251023217). Ethical approval was not required (9).

### 2.1 Literature search

We conducted comprehensive literature searches in PubMed, Embase, the Cochrane Library, and Web of Science for studies published from the inception of each database to January 27, 2025. The major search terms used in PubMed were as follows: “Heart Failure”, “Cardiac Failure”, “Congestive Heart Failure”, “Myocardial Failure”, “Controlling Nutritional Status score” and “CONUT” (Supplementary Table S1).

### 2.2 Study selection

To be considered for inclusion, studies had to fulfill the following selection criteria: Population: (1) Individuals (aged ≥18 years) with a confirmed diagnosis of heart failure; (2) All types of heart failure (HFrEF, HFmrEF, HFpEF); (3) Regardless of the New York Heart Association (NYHA) functional class.

Exposure: (4) Patients stratified into high and low CONUT score groups based on predefined cutoff values.

Outcomes: All-cause mortality, cardiovascular mortality, heart failure readmission rate and the composite outcomes of all-cause mortality or heart failure readmission; (5) Studies providing hazard ratio (HR) and 95% confidence interval (CI) data, either directly reported or calculable from available data; (6) Studies published in full-text form.

Studies were excluded based on the criteria listed below: (1) Review articles, case reports, conference abstracts, commentaries, and letters; (2) Studies not providing sufficient data to calculate HRs and 95% CIs; (3) Studies that did not report survival data; (4) Duplicate publications or those with overlapping data.

### 2.3 Data extraction and quality assessment

Two researchers, YY and ZPY, independently collected the data. Any inconsistencies were resolved through discussion with all co-authors. The extracted variables included the first author's name, year of publication, country or region of the study, study design, number of participants, patient age, patient type, LVEF, cut-off CONUT score, SMD and HRs (95% CIs) for outcomes (Table 2). The quality of the studies incorporated into the meta-analysis was assessed using the Newcastle-Ottawa Scale (NOS), which considers three domains for assessment: selection, comparability, and outcomes, with a maximum score of nine points. Studies scoring between 7 and 9 were classified as high quality (Table 3).

**Table 2:** Baseline characteristics of the included trials.

Author	Study period	Region	Study design	Population	No. of patients	Male	Female	Mean/median Age	Mean/median LVEF	CONUT cut-off	Calculation method for CONUT cutoff	Confounding factors	
												Statins	lipids
Nochioka 2013a	2006–2010	Japan	Prospective cohort	CHF	3,421	2,448	973	66.9	-	2	NA	No	No
Nochioka et al. (10)	2006–2010	Japan	Prospective cohort	CHF	3,421	2,448	973	66.9	-	2	NA	No	No
Nakagomi et al. (11)	2000–2011	Japan	Prospective cohort	CHF	114	85	29	66	26.6% ± 6.4%	3	NA	Yes	No
Agra et al. (35)	2014–2015	Spain	Retrospective cohort	HF	145	90	55	69	-	2	NA	Yes	No
Iwakami et al. (12)	2013–2015	Japan	Retrospective cohort	AHF	635	392	243	75	48.6	3	NA	Yes	No
Iwakami 2017b	2013–2015	Japan	Retrospective cohort	AHF	635	392	243	75	48.6	5	NA	Yes	No
Iwakami 2017c	2013–2015	Japan	Retrospective cohort	AHF	635	392	243	75	48.6	6	NA	Yes	No
La Rovere et al. (33)	2008–2010	Italy	Prospective cohort	CHF	466	401	65	61.3	33.7	1	NA	No	No
Hamada et al. (13)	2011–2014	Japan	Retrospective cohort	AHF	67	39	28	85.4	-	5	NA	No	No
Nishi et al. (14)	2012–2015	Japan	Retrospective cohort	HF	482	298	184	71.7	40.5	1	NA	No	No
Nishi 2018b	2012–2015	Japan	Retrospective cohort	HF	482	298	184	71.7	40.5	2	NA	No	No
Nishi 2018c	2012–2015	Japan	Retrospective cohort	HF	482	298	184	71.7	40.5	3	NA	No	No
Shirakabe et al. (15)	2000–2016	Japan	Retrospective cohort	AHF	458	302	156	76	40	4	The ROC curve	No	No
Shirakabe 2018b	2000–2016	Japan	Retrospective cohort	AHF	458	302	156	76	40	8	The ROC curve	No	No
Shirakabe 2018c	2000–2016	Japan	Retrospective cohort	AHF	458	302	156	76	40	9	The ROC curve	No	No
Sze et al. (29)	2000–2016	England	Prospective cohort	CHF	4,021	3,386	635	75	44	4	NA	No	No
Sze 2018b	2000–2016	England	Prospective cohort	CHF	4,021	3,386	635	75	44	8	NA	No	No
Sze 2018c	2000–2016	England	Prospective cohort	CHF	4,021	3,386	635	75	44	12	NA	No	No
Yoshihisa et al. (16)	2009–2015	Japan	Retrospective cohort	HF	1,307	792	515	66.5	42.2	-	NA	No	Yes
Chien et al. (23)	2012–2014	China	Retrospective cohort	AHF	1,120	441	679	77.2	-	-	NA	No	Yes
Kato 2020	2014–2016	Japan	Prospective cohort	AHF	2,466	1,412	1,054	≥70		4	NA	No	No
Komorita et al. (7)	2007–2013	Japan	Prospective cohort	CHF	506	277	229	71.6	62.7	5	The ROC curve	No	No
Uemura et al. (18)	2010–2014	Japan	Retrospective cohort	AHF	170	101	69	67.5	-	-		No	No
Sze et al. (30)	2016–2017	England	Prospective cohort	CHF	467	313	154	76	-	2	NA	No	No
Takada et al. (19)	2013–2019	Japan	Retrospective cohort	HF	1,705	1,099	606	71	40	2	NA	No	No
Liu et al. (24)	2020–2021	China	Prospective cohort	HF	402	267	135	61.7	-	2	NA	No	Yes
Uemura et al. (18)	2016–2018	Japan	Retrospective cohort	AHF	465	268	197	74.8	-	2	NA	No	No
Agnoletti et al. (34)	2013–2015	Italy	Prospective cohort	AHF	293	141	152	83.7	-	4	NA	No	No
Chen et al. (25)	2017–2019	China	Retrospective Cohort	CHF	371	260	111	88	-	4	The ROC curve	No	No
Iida et al. (21)	2014–2021	Japan	Prospective cohort	CHF	1,617	906	711	78.6	-	4	NA	No	No
Iida 2023b	2014–2021	Japan	Prospective cohort	CHF	1,617	906	711	78.6	-	8	NA	No	No
Iida 2023c	2014–2021	Japan	Prospective cohort	CHF	1,617	906	711	78.6	-	9	NA	No	No
Lin 2023a	2008–2018	China	Retrospective Cohort	HF	1,371	814	557	72	45	4	NA	No	No
Lin 2023b	2008–2018	China	Retrospective Cohort	HF	1,371	814	557	72	45	8	NA	No	No
Lin 2023c	2008–2018	China	Retrospective Cohort	HF	1,371	814	557	72	45	12	NA	No	No
Zhao et al. (8)	2016–2021	China	Retrospective Cohort	CHF	187	132	55	66.9	32.5	-	The ROC curve	No	No
Fan et al. (27)	2016–2021	China	Retrospective Cohort	CHF	218	147	71	85	56	2	The ROC curve	Yes	No
Fan 2024b	2016–2021	China	Retrospective Cohort	CHF	187	132	55	66.9	32.5	4	The ROC curve	Yes	No
Hikoso 2024	2016–2020	Japan	Prospective cohort	CHF	547	255	292	82	-	2	The ROC curve	No	No
Huang et al. (28)	2019–2022	China	Retrospective Cohort	AHF	1,230	724	506	68	-	4	NA	Yes	Yes
Huang 2024b	2019–2022	China	Retrospective Cohort	AHF	1,230	724	506	68	-	8	NA	Yes	Yes
Huang 2024c	2019–2022	China	Retrospective Cohort	AHF	1,230	724	506	68	-	12	NA	Yes	Yes
Prokopidis et al. (31)	1999–2018	America	Retrospective Cohort	HF	1,501	844	657	70	-	2	NA	Yes	No
Prokopidis 2025b	1999–2018	America	Retrospective Cohort	HF	1,501	844	657	70	-	12	NA	Yes	No
Zhang et al. (32)	1999–2018	America	Retrospective Cohort	HF	1,232	719	513	68	-	-	NA	No	No

**Table 3:** Quality evaluation of the eligible studies with Newcastle–Ottawa scale.

Study	Selection				Comparability		Outcome		
	Representative-ness	Selection of non-exposed	Ascertainment of exposure	Outcome not present at start	Comparability on most important factors	Comparability on other risk factors	Assessment of outcome	Long enough follow-up (media*n* ≥ 3 months)	Adequacy (completeness) of follow-up
Nochioka, K et al. (10)	-	*	*	*	-	-	*	*	*
Nakagomi, A et al, (11)	-	*	*	*	-	-	*	*	*
Agra Bermejo, R et al. (35)	*	*	*	*	-	-	*	*	*
Iwakami, N et al. (12)	-	*	*	*	-	-	*	*	*
La Rovere, M et al. (33)	*	*	*	*	-	-	*	*	*
Hamada, T et al. (13)	-	*	*	*	-	-	*	*	*
Nishi, I e al. (14)	-	*	*	*	*	*	*	-	*
Shirakabe, A et al. (15)	*	*	*	*	-	-	*	-	*
Sze, S et al. (29)	*	*	*	*	-	-	*	*	*
Yoshihisa, A et al. (16)	*	*	*	*	*	-	*	*	*
Chien, S et al. (23)	-	*	*	*	-	-	*	*	*
Kato, T et al. (7)	*	*	-	*	-	-	*	-	-
Komorita, T et al. (17)	*	*	*	*	-	-	*	*	*
Uemura, Y et al. (18)	-	*	*	*	-	-	*	*	*
Sze, S et al. (30)	*	*	*	*	-	-	*	*	*
Takada, T et al (19)	*	*	*	*	-	-	*	*	*
Liu, J et al. (24)	-	*	*	*	-	-	*	*	*
Uemura, Y et al. (20)	*	*	*	*	-	-	*	*	*
Agnoletti, D et al. (34)	-	*	*	*	-	-	*	*	*
Chen, Y et al. (25)	*	*	*	*	-	-	*	*	*
Iida, Y et al. (21)	-	*	*	*	-	-	*	*	*
Liang, L et al. (26)	*	*	*	*	-	-	*	*	*
Zhao, J et al. (8)	-	*	*	*	-	-	*	-	*
Fan, X et al. (27)	*	*	*	*	-	-	*	*	*
Hikoso, S 2024	-	*	*	*	-	-	*	*	*
Huang, X et al. (28)	*	*	*	*	-	-	*	*	*
Prokopidis, K et al. (31)	-	*	*	*	-	-	*	*	*
Zhang, F et al. (32)	-	*	*	*	-	-	*	*	*

*indicates criterion met; - indicates significant of criterion not met.

### 2.4 Statistical analysis

The statistical analyses were performed using RevMan 5.4 and STATA 15. HRs or standardized mean differences (SMD) were employed as effect sizes for categorical and continuous variables, respectively, with corresponding 95% CIs calculated. We evaluated heterogeneity using Cochran's *Q* test and I² statistic. A *P*-value below 0.10 or an I² above 50% indicated significant heterogeneity, prompting the use of a random-effects model for the meta-analysis. Statistical significance was set at a two-sided *P*-value of less than 0.05. Publication bias was evaluated using Egger's test alongside a visual inspection of funnel plots. Sensitivity analyses were conducted to assess the stability of the meta-analytic results and to investigate possible contributors to heterogeneity. Subgroup analyses were conducted according to study design, age, geographic region, heart failure subtype, and CONUT score cut-off values to investigate clinical and methodological heterogeneity.

## 3 Results

### 3.1 Search results

**Figure 1:**
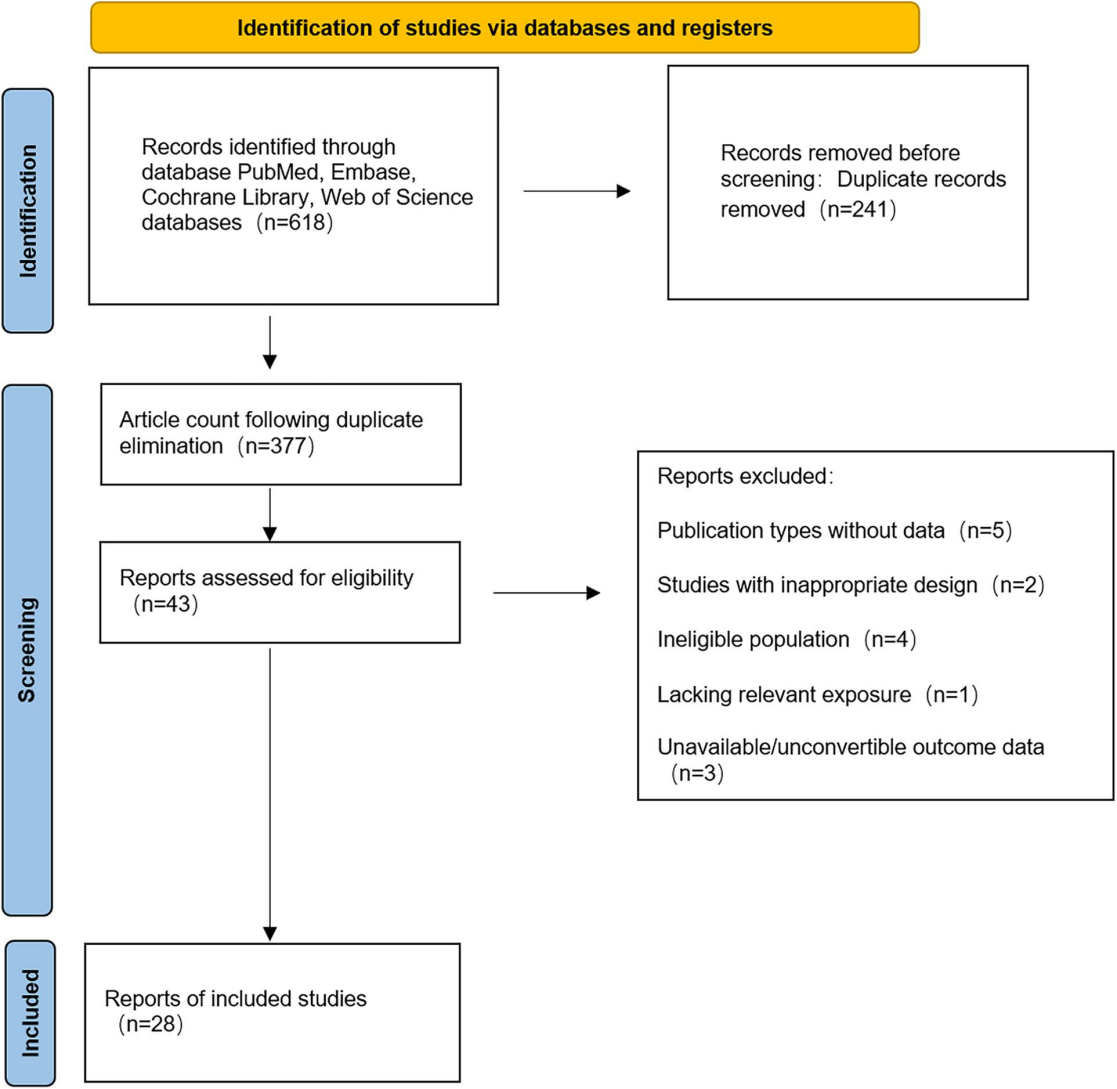
Literature search flowchart.

A total of 618 records were retrieved from PubMed (*n* = 166), EMBASE (*n* = 249), Cochrane Library (*n* = 7) and Web of Science (*n* = 196). Following the removal of duplicates using EndNote X9 (241 duplicates excluded), 377 articles remained for screening. Following the screening of titles and abstracts, 43 articles were selected for full-text review, with 28 studies (7, 10–22) ultimately meeting the inclusion criteria. The geographical distribution of the 28 eligible studies was as follows: Japan (7, 10–22) (*n* = 14), China (8, 23–28) (*n* = 7), United Kingdom (29, 30) (*n* = 2), United States (31, 32) (*n* = 2), Italy (33, 34) (*n* = 2), and Spain (35) (*n* = 1). The study designs comprised 17 retrospective cohort studies (8, 10, 13–17, 20–22, 24, 26–29) and 11 prospective cohort studies (7, 10, 11, 17, 21–23, 29, 30, 33–35). All included cohort research articles, published in English, appeared between 2013 and 2025. The retrieval flowchart is presented in Figure 1.

### 3.2 Study quality

Each of the 28 included studies received a score ranging from 6–8 on the NOS, indicating high methodological quality (Table 3).

### 3.3 Meta-analysis results

#### 3.3.1 All-cause mortality

**Figure 2:**
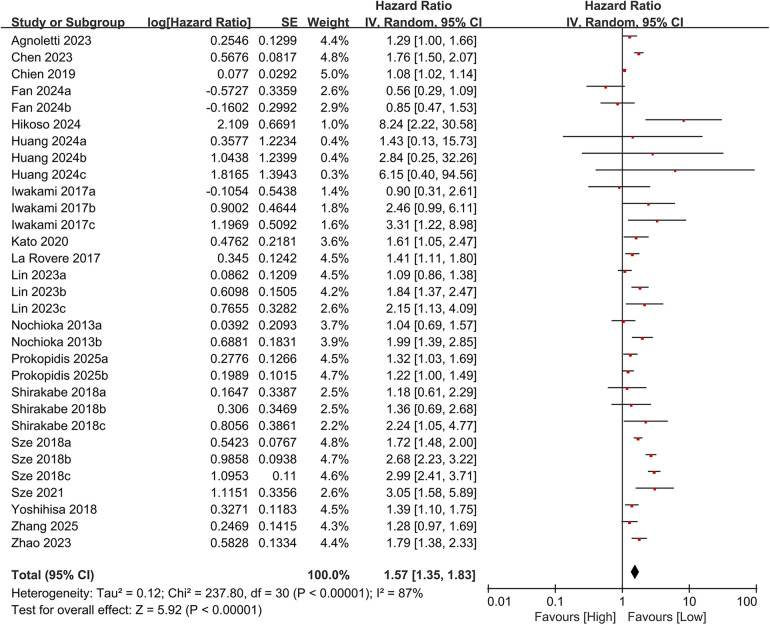
Forest plot of all-cause mortality (categorical variable).

**Figure 3:**
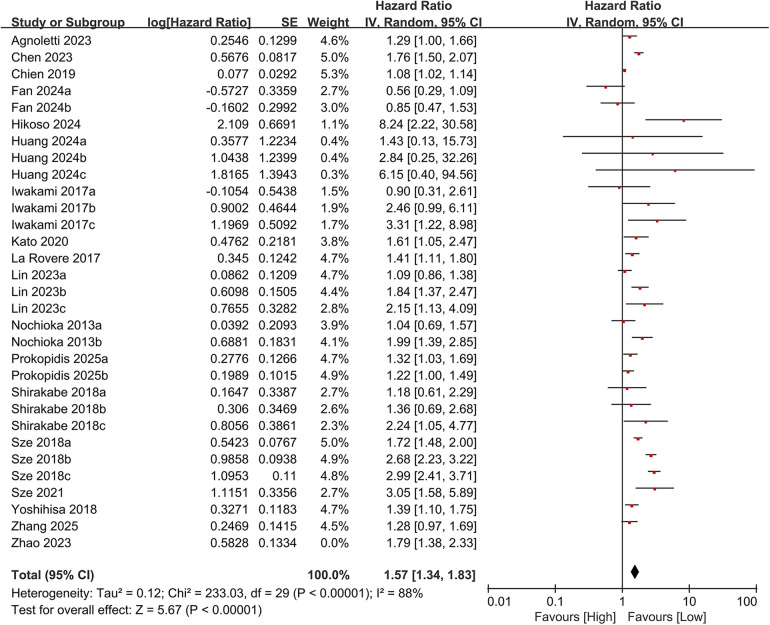
Forest plot for all-cause mortality after sensitivity analysis (categorical variable).

**Figure 4:**
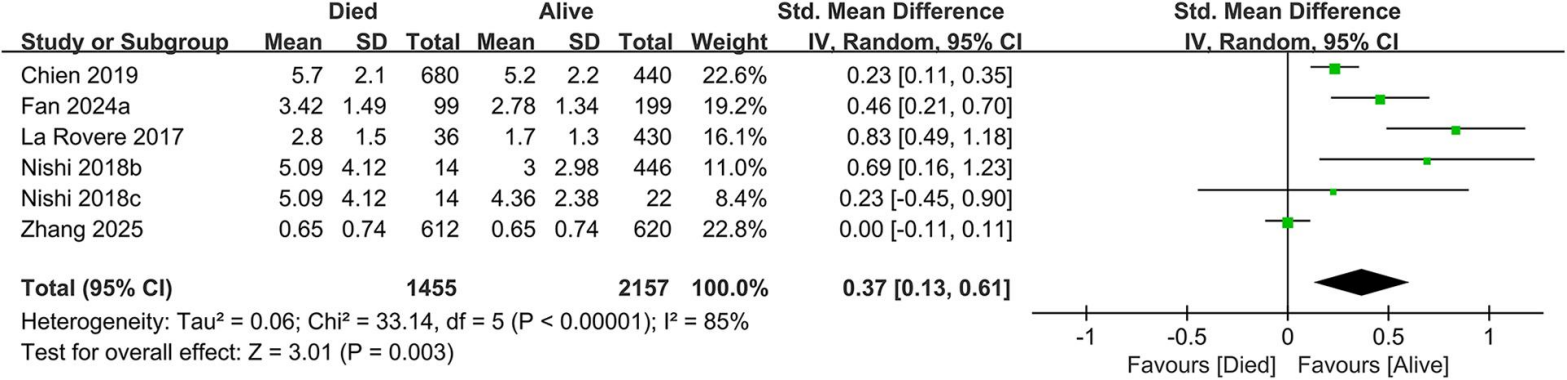
Forest plot of all-cause mortality (continuous variable).

**Figure 5:**
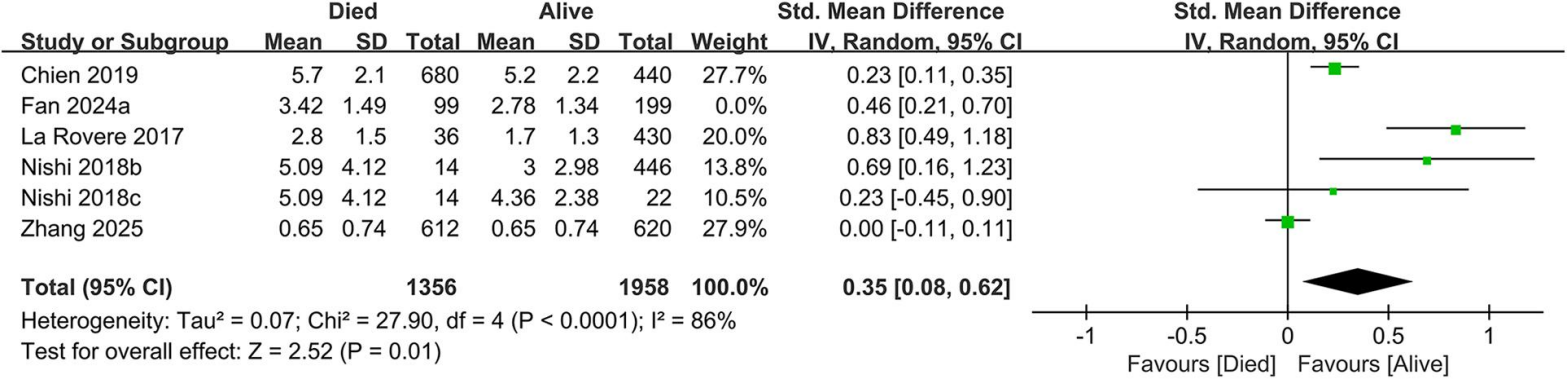
Forest plot for all-cause mortality after sensitivity analysis (continuous variable).

In total, 19 studies evaluated the link between CONUT scores and all-cause mortality. Of these, 17 studies analyzed CONUT as a categorical variable. The meta-analysis demonstrated that elevated CONUT scores were significantly correlated with a higher risk of all-cause mortality (HR = 1.57, 95% CI 1.35–1.83; *P* < 0.00001), with substantial heterogeneity across studies (*P* < 0.00001, I² = 87%; Figure 2). Sensitivity analyses were subsequently conducted to explore potential sources of heterogeneity. Sequential exclusion of individual studies yielded stable pooled HR estimates, ranging from 1.52–1.62, with all recalculated estimates maintaining overlapping confidence intervals with the original analysis, indicating the robustness of the primary findings (Figure 3). Notably, the high CONUT group demonstrated significantly elevated risk compared to the low CONUT group. Six studies evaluated CONUT as a continuous variable, revealing a 37% mean increase in CONUT scores among deceased patients vs. survivors (SMD = 0.37, 95% CI 0.13–0.61; *P* = 0.003; Figure 4). Despite significant statistical heterogeneity (I² = 85%), sensitivity analyses confirmed the reliability of the CONUT score–mortality association, with pooled effect sizes remaining stable (range: 0.35–.47). All recalculated estimates exhibited overlapping confidence intervals with the original analysis, further supporting the robustness of the primary results (Figure 5).

#### 3.3.2 Cardiovascular mortality

**Figure 6:**
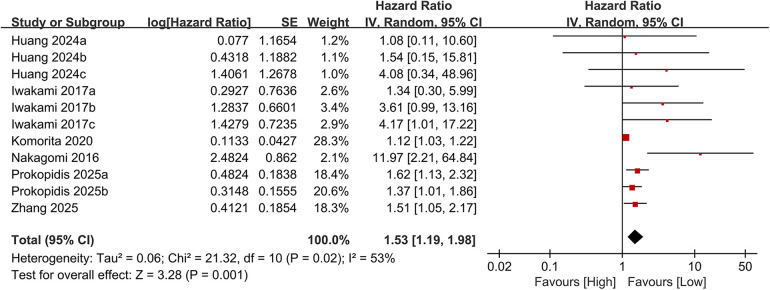
Forest plot of cardiovascular mortality.

**Figure 7:**
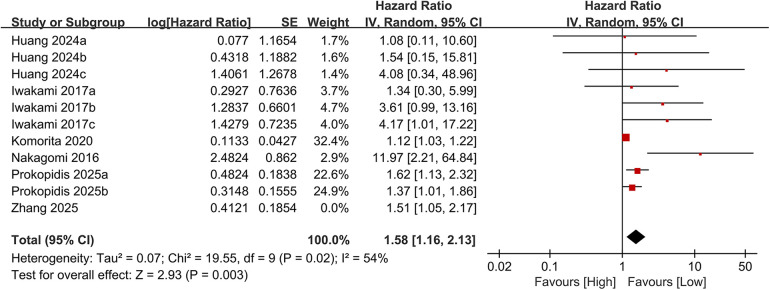
Forest plot for cardiovascular mortality after sensitivity analysis.

Eleven studies examined the association between the CONUT score and cardiovascular mortality. When analyzed as a categorical variable, the meta-analysis revealed a significantly elevated risk of cardiovascular mortality in the high CONUT group (HR = 1.53, 95% CI 1.19–1.98; *P* = 0.001), with moderate heterogeneity across studies (*P* = 0.001, I² = 53%; Figure 6). Sensitivity analyses, based on the sequential exclusion of individual studies, confirmed the robustness of the observed relationship between CONUT scores and cardiovascular mortality (Figure 7). Despite methodological heterogeneity, all analytical scenarios consistently demonstrated that worsening nutritional status significantly increased the risk of cardiovascular mortality.

#### 3.3.3 Heart failure readmission rate

**Figure 8:**
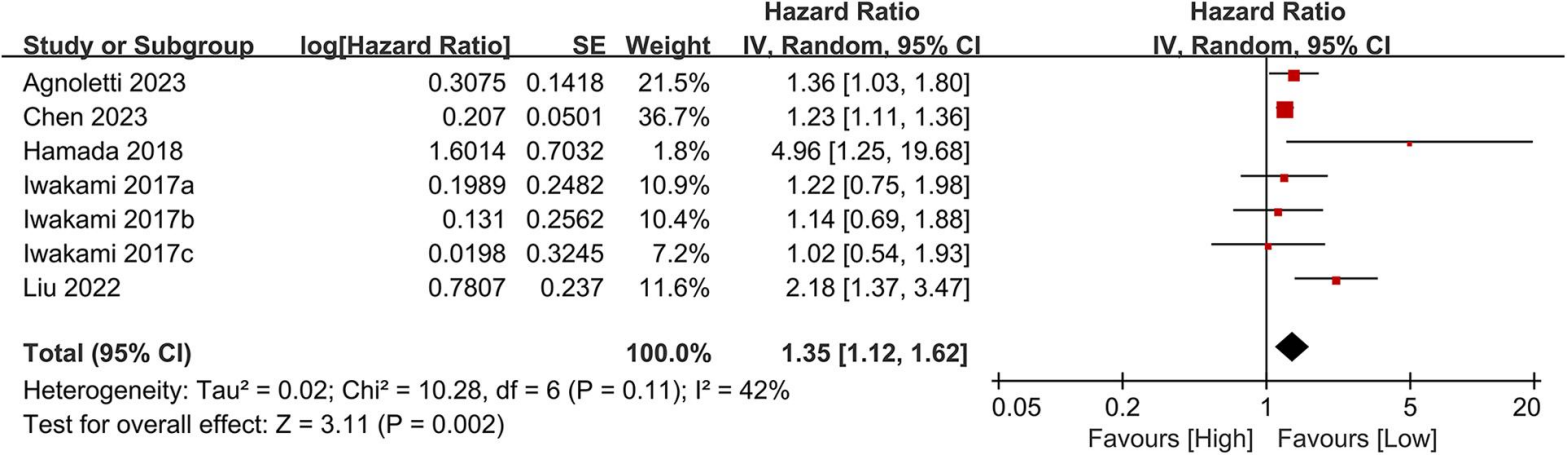
Forest plot of heart failure readmission rate.

**Figure 9:**
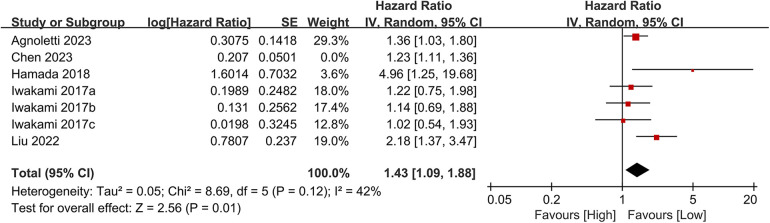
Forest plot for heart failure readmission rate after sensitivity analysis.

Seven studies examined the association between CONUT scores and heart failure readmission rates. The meta-analysis indicated a significant link between high CONUT scores and increased heart failure readmission risk (HR = 1.35, 95% CI 1.12–1.62; *P* = 0.02), with moderate heterogeneity across studies (*P* = 0.002, I² = 42%; Figure 8). To assess the stability of the results, sensitivity analyses were performed by sequentially excluding individual studies. All recalculated estimates showed overlapping confidence intervals with the original analysis, further confirming the robustness of the primary findings (Figure 9).

#### 3.3.4 Composite outcomes

**Figure 10:**
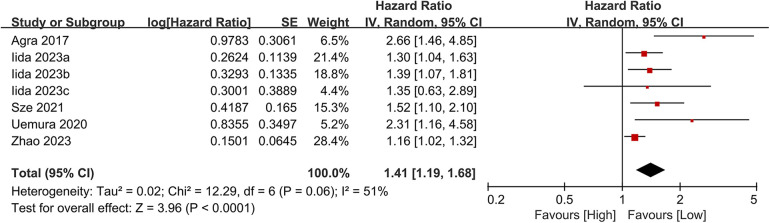
Forest plot of composite outcome.

**Figure 11:**
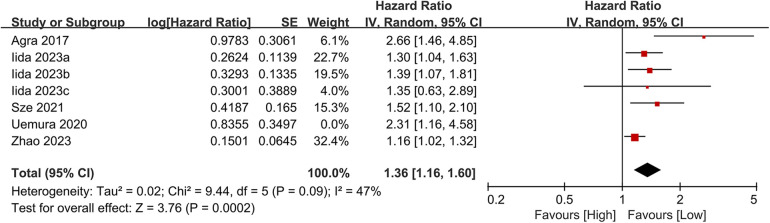
Forest plot for the composite outcome after sensitivity analysis.

Seven studies investigated the association between the CONUT score and the composite outcome of all-cause mortality or heart failure readmission. The meta-analysis demonstrated a significant association between elevated CONUT scores and increased risk of the composite outcome (HR = 1.41, 95% CI 1.19–1.68; *P* < 0.00001), with moderate heterogeneity across studies (*P* < 0.00001, I² = 51%; Figure 10). Sensitivity analyses were conducted to assess the stability of the results, involving the sequential exclusion of individual studies. All recalculated pooled estimates exhibited overlapping confidence intervals with the original analysis, confirming the robustness of the primary findings (Figure 11).

#### 3.3.5 Publication bias

**Figure 12:**
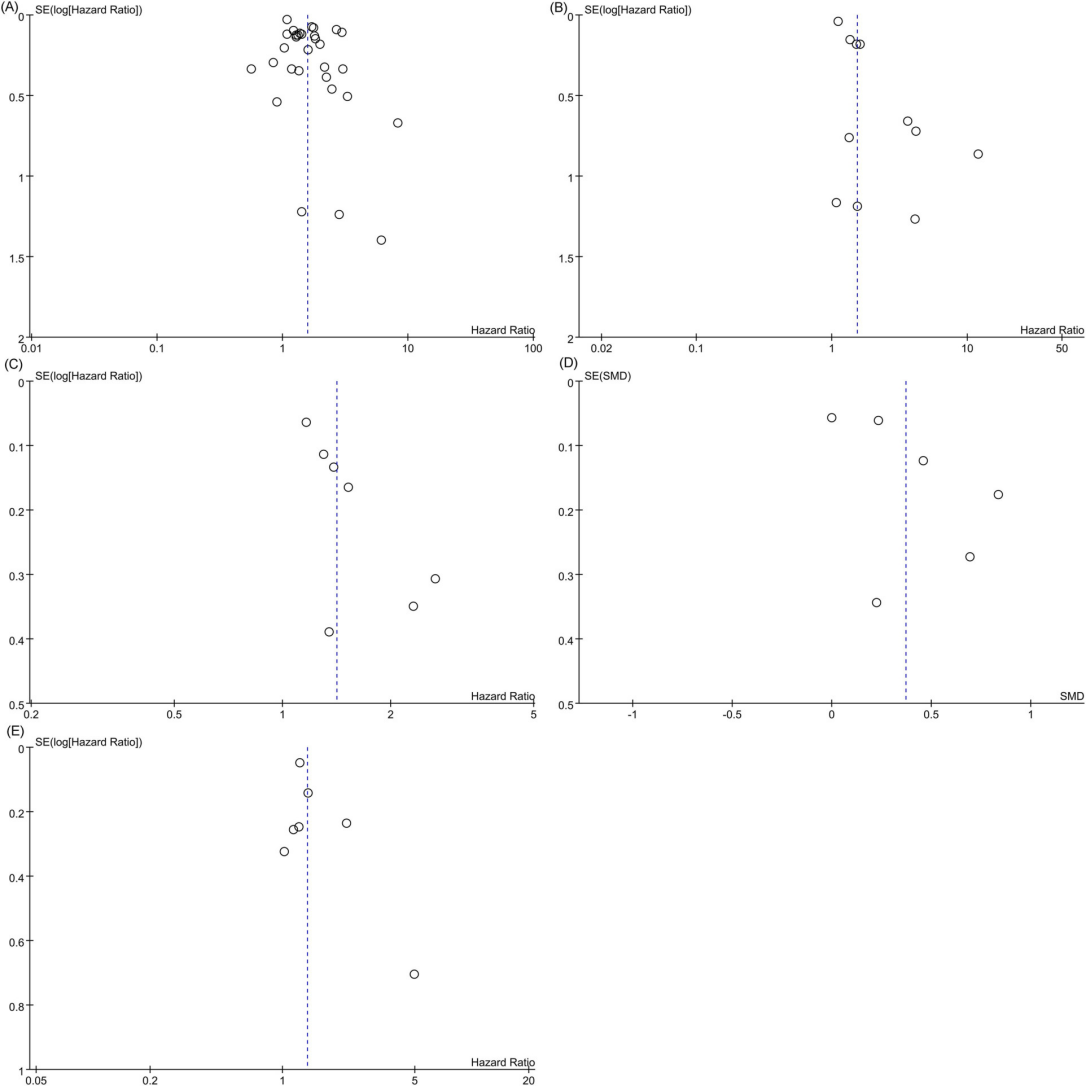
Funnel plot. **(A)** All-cause mortality (categorical variable); **(B)** Cardiovascular mortality; **(C)** Funnel plot for the composite outcome; **(D)** Funnel plot of all-cause mortality (continuous variable); **(E)** Funnel plot of heart failure readmission rate.

Egger's regression test was used to evaluate publication bias. Evidence of significant publication bias was found in the meta-analyses of all-cause mortality (categorical variable analysis), cardiovascular mortality, and the composite outcome of all-cause mortality or heart failure readmission (Egger's test: *P* = 0.022, *P* = 0.004, and *P* = 0.013). Visual inspection of the corresponding funnel plots revealed asymmetric distributions (Figures 12A–C). In contrast, Egger's test demonstrated no significant publication bias in the meta-analyses of all-cause mortality (continuous variable analysis) or heart failure readmission rates (*P* = 0.129 and *P* = 0.247, respectively). Their funnel plots exhibited approximate symmetry with evenly distributed data points, indicating a low risk of publication bias (Figures 12D,E). The consistency between funnel plot observations and Egger's test results further validated the reliability of these conclusions.

#### 3.3.6 Subgroup analysis

Subgroup analyses based on study design, age, region, CONUT cutoff value, heart failure type, and NOS score demonstrated that a higher CONUT score remained significantly associated with an increased risk of all-cause mortality across all predefined subgroups. The predictive value was consistent regardless of the specific optimal cutoff value applied. With respect to cardiovascular mortality, a significant association was observed only when the CONUT cutoff exceeded 9, suggesting that severe malnutrition may elevate the risk of cardiovascular death. Furthermore, in patients with acute heart failure (AHF), elevated CONUT scores were positively correlated with both all-cause and cardiovascular mortality. In contrast, among patients with chronic heart failure (CHF), the CONUT score remained a significant predictor of all-cause mortality, although its association with cardiovascular mortality did not reach statistical significance, In Asian and European populations, a higher CONUT score was associated with a significantly increased risk of all-cause mortality. Whereas in America, this association was not statistically significant, possibly due to the limited number of studies available (Table 4).

**Table 4:** Subgroup analysis of All-cause mortality and cardiovascular mortality.

Subgroup	All-cause mortality_Study group	All-cause mortality_HR [95% CI]	All-cause mortality_*P* value	All-cause mortality_I2	Cardiovascular mortality_Study group	Cardiovascular mortality_HR [95% CI]	Cardiovascular mortality_*P* value	Cardiovascular mortality_I2
Total	18	1.57 [1.35–1.83]	<0.00001	0.87	6	1.53[1.19–1.98]	0.001	0.53
Study design								
Prospective	7	1.91 [1.50–2.44]	<0.00001	0.86	2	3.31[0.13–31.27]	0.33	0.78
Retrospective	11	1.38 [1.20–1.60]	<0.00001	0.74	4	1.54 [1.28–1.86]	<0.00001	0
Mean/median age								
≥70 y	12	1.61 [1.33–1.95]	<0.00001	0.91	3	1.41 [1.08–1.84]	0.01	0.55
<70 y	6	1.47 [1.28–1.69]	<0.00001	0.22	3	2.31 [0.98–5.41]	0.05	0.35
Region								
Asia	12	1.48 [1.25–1.75]	<0.00001	0.78	4	2.30 [1.15–4.61]	0.02	0.53
Europe	4	1.82 [1.34–2.48]	<0.00001	0.96	0			
America	2	1.31 [1.09–1.57]	0.005	0.86	2	1.48 [1.22–1.80]	<0.00001	0
CONUT cut-off								
2–4	13	1.41 [1.18–1.68]	0.0001	0.70	4	2.12 [0.90–5.00]	0.09	0.45
5–8	5	2.22 [1.73–2.85]	<0.00001	0.34	4	1.94[0.86–4.37]	0.11	0.54
9–12	5	2.11 [1.19–3.73]	0/01	0/89	2	1.39[1.03–1.88]	0.03	0
Types of Heart Failure								
Chronic Heart Failure	10	1.76 [1.42–2.19]	<0.00001	0.85	1	3.13[0.31–31.27]	0.33	0.87
Acute Heart Failure	6	1.38 [1.13–1.70]	0.002	0.41	2	2.53[1.26–5.06]	0.009	0
NOS								
≥6	16	1.56 [1.33–1.83]	<0.00001	0.88	0			
<6	2	1.74 [1.39–2.17]	<0.00001	0	0			

## 4 Discussion

The aim of this meta-analysis was to evaluate the prognostic value of the CONUT score in individuals with heart failure, drawing on data from 28 cohort studies encompassing a total of 26,984 participants. This meta-analysis showed that individuals with elevated CONUT scores faced significantly increased risks of all-cause mortality, cardiovascular mortality, and heart failure readmission. The underlying mechanisms may involve malnutrition-driven pathological cascades, including inflammatory activation, immune dysfunction, and metabolic dysregulation. For instance, hypoalbuminemia reflects protein-energy malnutrition, decreased total cholesterol indicates impaired lipid metabolism, and lymphocytopenia correlates with immune exhaustion-factors that synergistically contribute to accelerated cardiac decompensation.

The study by Li et al. (36) investigated the prognostic value of the CONUT score for all-cause mortality in patients with heart failure. Their results demonstrated that patients with CONUT scores ≥2 had a 1.92-fold increased risk of all-cause mortality compared to those with scores 0–1 (RR = 1.92, 95% CI 1.58–2.34). Subgroup analysis further revealed a 1.78-fold elevated mortality risk in the malnourished subgroup (RR = 1.78, 95% CI 1.29–2.46). Unlike the meta-analysis by Li et al., which highlighted the CONUT score's predictive value for all-cause mortality and identified a stronger association in CHF patients, our subgroup analyses revealed no statistically significant relation between CONUT and cardiovascular mortality in CHF populations (*P* > 0.05). This difference could be explained by the relatively smaller sample size in the CHF subgroup or the potential masking of the independent prognostic effect of nutritional status by overriding pathophysiological mechanisms in CHF, such as hemodynamic deterioration and neurohormonal activation. In addition, we performed subgroup analyses based on different optimal cutoff values of the CONUT score. Regardless of the specific cutoff value used, a higher CONUT score was consistently associated with a significantly increased risk of all-cause mortality. Regarding the prediction of cardiovascular mortality, a significant association was observed only when the cutoff value was set above 9, suggesting that severe malnutrition may increase the risk of cardiovascular death. Among the studies included in this meta-analysis, five used ROC analysis to determine the optimal CONUT cutoff value in patients with CHF, which were reported as 2, 2.5, and 4, respectively. One study identified a cutoff of 5 for patients with AHF. In older patients over 70 years of age, cutoff values ranging from 2–5 were shown to effectively predict clinical outcomes. Differences in nutritional-metabolic status and disease severity across patient phenotypes may account for the variation in optimal thresholds. Therefore, further studies targeting specific patient subgroups are warranted to validate phenotype-specific CONUT cutoff values.

In comparison to other nutritional screening and assessment tools, the CONUT score is calculated using objective laboratory parameters, including serum albumin levels and lymphocyte counts (37), providing a more standardized reflection of nutritional status with minimized subjective bias. For instance, the Subjective Global Assessment (SGA) primarily relies on clinicians' qualitative evaluations of dietary intake, weight changes, and gastrointestinal symptoms, which may introduce inter-rater variability (38). The validity of the CONUT score may be influenced by factors such as lipid-lowering medications and infections. Six studies adjusted for statin use in their multivariate Cox regression models, and four studies adjusted for blood lipid profiles. The results demonstrated that the CONUT score remained a significant predictor of both all-cause mortality and cardiovascular mortality even after adjusting for these and other important clinical confounders. However, significant heterogeneity exists in the CONUT cutoff values (range: 2–5 points) across current studies, highlighting the need for large-scale cohort studies to establish population-specific optimal thresholds. While the European Society for Clinical Nutrition and Metabolism (ESPEN) recommends a cutoff ≥4 points as diagnostic for malnutrition (37), this standard still requires validation across diverse ethnic and clinical populations. Further mechanistic studies using animal models are warranted to elucidate how malnutrition directly exacerbates myocardial injury-for example, investigating whether hypoalbuminemia potentiates cardiomyocyte death via autophagy dysregulation or other molecular pathways.

The precise mechanisms underlying the prognostic value of the CONUT score in HF remain incompletely elucidated. Serum albumin synthesis is influenced by nutritional intake and systemic inflammation (39). Several studies (40) have reported that hypoalbuminemia correlates with adverse clinical outcomes in HF patients, with inflammation and malnutrition posited as primary etiological contributors. Albumin exerts antioxidant and anti-inflammatory properties, and its deficiency may impair myocardial repair capacity, accelerating ventricular remodeling. Cholesterol levels, reflective of nutritional status, also impact prognosis. Extensive evidence confirms the association between hypercholesterolemia and coronary artery disease (CAD) progression and mortality risk (41). Data from the Framingham Study indicate that elevated serum cholesterol not only jeopardizes cardiovascular health but also serves as a significant risk factor for HF development (42). Pathophysiologically, hypocholesterolemia may signal two underlying states: (1) neurohormonal overactivation disrupting cholesterol metabolism, or (2) hypermetabolic stress exacerbating nutrient depletion. Furthermore, hypocholesterolemia is closely linked to malnutrition and cachexia-both strongly associated with poor HF outcomes (43). Inflammation is an important factor in HF pathogenesis and progression (44). Chronic inflammation and malnutrition in HF patients drive T-cell exhaustion, increasing susceptibility to infections (e.g., pneumonia, sepsis), which frequently trigger acute decompensation. Lymphocytopenia (<1,500/mm³) has been linked to an 82% higher mortality risk in HF patients, independent of ejection fraction (45). Malnutrition activates the nuclear factor kappa-B (NF-κB) pathway, promoting the release of proinflammatory cytokines such as tumor necrosis factor-alpha (TNF-α) and interleukin-6 (IL-6), which induce myocardial fibrosis and microvascular dysfunction. Concurrently, metabolic disturbances (e.g., insulin resistance, lipid peroxidation) exacerbate mitochondrial dysfunction, leading to cardiomyocyte energy depletion and apoptosis (46). These interconnected mechanisms synergistically accelerate cardiac functional decline and adverse clinical outcomes.

This study presents some limitations. First, the overall quality of the evidence may be limited by the inclusion of some studies with relatively low methodological quality, as two studies scored below 6 on the Newcastle–Ottawa Scale (NOS). However, subgroup and sensitivity analyses based on NOS scores showed no significant change in heterogeneity, and the association between malnutrition and all-cause mortality remained statistically significant, which strengthens the robustness of our primary conclusion. Second, publication bias was detected for certain outcomes, possibly due to the inclusion of several small-sample studies and the absence of gray literature. This bias may have led to a slight overestimation of the predictive value of the CONUT score. Nevertheless, sensitivity and subgroup analyses indicated that the overall results are robust, supporting the clinical value of the CONUT score as a tool for assessing nutritional status and prognosis in patients with heart failure. Future high-quality, large-scale, prospective studies are needed to further validate its cutoff values and clinical applicability. Third, the included studies used different cutoff values for the CONUT score (e.g., ≥2 vs. ≥5), which may have introduced heterogeneity into the results. Fourth, this study did not assess the potential influence of nutritional indicators—such as acute infection, use of lipid-lowering agents, or SGLT2 inhibitors—on the CONUT score. Further research is warranted to explore the relationship between medication dosage and the individual components of the CONUT score. Fifth, due to limited data availability, original individual-level data from the included articles could not be obtained. Therefore, a detailed dose-response analysis quantifying the specific impact of each 1-point increase in the CONUT score on prognostic risk was not feasible. Future studies should consider conducting individual participant data meta-analyses or large-scale cohort studies to further explore this dose-response relationship, thereby addressing the current gaps and providing direction for subsequent research. Finally, as the majority of included studies were observational in design, a causal relationship between the CONUT score and prognosis cannot be established.

## 5 Conclusion

Based on the CONUT score definition, malnutrition is recognized as an effective predictor of all-cause mortality in HF patients after adjusting for confounders. Utilizing the CONUT score for nutritional assessment enables clinicians to identify HF patients at elevated risk for adverse clinical outcomes. Acknowledging these limitations, prospective, large-scale studies are warranted to further validate the prognostic relevance of the CONUT score across diverse HF populations.

## Data Availability

The original contributions presented in the study are included in the article/Supplementary Material, further inquiries can be directed to the corresponding author.
