# Supplementary material for: Association between controlling nutritional status score and the prognosis of patients with heart failure: a systematic review and meta-analysis
Source: Front Cardiovasc Med. 2025 Oct 20;12:1665713. doi: 10.3389/fcvm.2025.1665713 (PMC12580275; doi:10.3389/fcvm.2025.1665713)
Supplement: Supplementary file 1 [file Table1.pdf]

# *Supplementary Material*

**Table S1** Search strategy table

| Search Strategy | Taking PubMed as an example |
|---|---|
| #1 | ("Heart Failure"[Mesh]) OR (((Cardiac Failure) OR (Congestive Heart Failure)) OR (Myocardial Failure)) |
| #2 | (Controlling Nutritional Status score) OR (CONUT) |
| #3 | #1 AND #2 |
